# An Unusual Case of Gullo's Syndrome Concomitant with Serious Endometriosis Disease in a Postmenopausal Woman

**DOI:** 10.1155/2018/6310245

**Published:** 2018-06-24

**Authors:** Abdelhakim Ainahi, Abdellaziz Ziane, Lahcen Wakrim, Naima Elmdaghri, Abdelhamid Barakat

**Affiliations:** ^1^Hormonology and Tumor Markers Laboratory, Institut Pasteur du Maroc, Casablanca, Morocco; ^2^Private Practice, Casablanca, Morocco; ^3^Virology Unit, Immunovirology Laboratory, Institut Pasteur du Maroc, Casablanca, Morocco; ^4^Human Molecular Genetics Laboratory, Institut Pasteur du Maroc, Casablanca, Morocco

## Abstract

Gullo's syndrome is a singular physiological phenomenon defined by an abnormal increase in serum pancreatic enzyme levels that may occur in healthy subjects in the absence of pancreatic disorders. During routine health examination in a 54-year-old postmenopausal woman with severe endometriosis, elevated values of serum amylase and lipase were fortuitously observed (198 and 1461 U/L, resp.). Over five years of regular pancreas surveillance, all clinical, biological, and imaging investigations were normal. However, the pancreatic enzyme levels have shown considerable fluctuations including some episodic transient normalization. The description of this benign pancreatic hyperenzymemia case incidentally associated with endometriosis disease is a very rare clinical situation. More in-depth documentation of this phenomenon may help clinicians to avoid unnecessary diagnostic management approaches and reassure the concerned patients that this affection would not be so worrying.

## 1. Introduction

An abnormal increase in serum pancreatic enzyme levels is usually associated with pancreatic disorders [1]. However, several other nonpancreatic diseases were demonstrated to be related with increasing levels of pancreatic enzymes. Among these pathologies, the most frequent are ectopic pregnancy, macroamylasemia, acute cholecystitis, virus infection, hypertriglyceridemia, intestinal infarction, duodenal ulcer, obstructive bowel disorders, liver diseases, and renal insufficiency [2]. In 1996, Gullo described a new syndrome, named “Gullo's syndrome,” characterized by persistent high levels of serum pancreatic enzymes in the absence of any pancreatic alterations [3].

Endometriosis is a chronic gynecological disease that affects about 10% to 15% of women of reproductive age [4, 5]. In this pathology, endometrial tissues were found in some other unusual distant organs including the gastrointestinal tract, pancreas, and kidneys. However, the exact pathophysiology of this disease remains undetermined. Prevailing reports hypothesized multifactorial mechanisms, including inflammation, retrograde menstruation, fluctuating levels of estrogen and progesterone, immune dysfunction, apoptosis suppression, or genetic variations [5–7].

To the best of our knowledge, only a few cases of Gullo's syndrome have been previously documented in the literature. Interestingly, no previous description of endometriosis disease cases concomitant with benign pancreatic hyperenzymemia has been reported so far. We herein focused on possible incidental association between Gullo's syndrome and endometriosis disease in Moroccan postmenopausal woman.

## 2. Case Report

**Figure 1:**
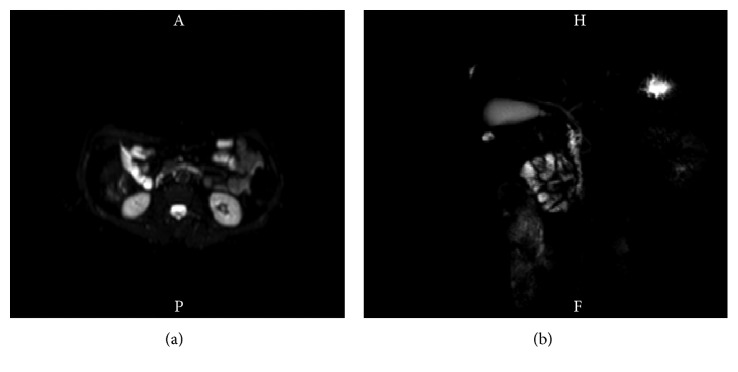
Normal representative magnetic resonance imaging of the (a) abdomen and (b) bladder and bile duct.

**Figure 2:**
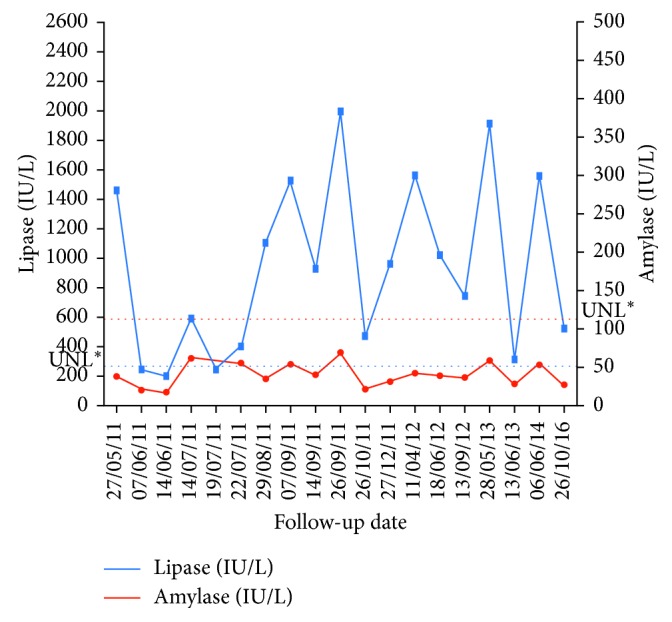
Serial follow-up of serum lipase and amylase levels in postmenopausal women. ^*∗*^Upper normal limit.

A 54-year-old postmenopausal woman from Casablanca (Morocco), presenting episodic abdominal complaints, was referred to our outpatient institution for laboratory testing as part of a routine checkup in May 2011. Her medical history was significant for extensive endometriosis confirmed by the laparoscopy procedure and infertility. The patient had surgery followed by hormonal therapy in 2003. At initial blood testing, the results indicated normal cell count with a hemoglobin of 14 gm/dl, a total leukocyte count of 8,200/mm, and a platelet count of 3,44,000/mm. Serum urea, creatinine, bilirubin, transaminases, total cholesterol, triglycerides, calcium, fasting blood glucose, C-reactive protein, gamma-glutamyl transpeptidase, alkaline phosphatase levels, and other electrolytes were normal (Table 1). However, the serum amylase level was 198 IU/L (reference range 30–110), and serum lipase increased to reach 1461 IU/L which is fivefold over the upper limit (reference range 27–280). Blood pancreatic isoamylase values were also abnormal. As the abnormal pancreatic enzyme secretions usually denote pancreatic pathology, a deep screening for pancreatic alterations was done to find out the possible causes of the elevated levels of lipase and amylase. Serological tests for hepatitis A, B, and C viruses and human immunodeficiency virus (HIV) were negative. The tumor markers (carcinoembryonic antigen, carbohydrate antigen 19-9, alpha-fetal protein, and carbohydrate antigen 125) showed normal ranges (Table 1). To exclude the common sources of hyperlipasemia and hyperamylasemia such as macroamylasemia, autoimmune diseases like systemic lupus erythematosus, celiac disease, and inflammatory bowel, investigations were done and all were absolutely normal. Abdominal ultrasonography and magnetic resonance imaging scans were performed in 2011 and showed normal pancreas, liver, and biliary tree (Figure 1). The patient denied weight loss, diarrhea, vomiting, cigarette smoking, or alcohol drinking. The biochemical screening of pancreatic hyperenzymemia was negative in the patient's family members. Given the interest of this case, we decided to follow-up the patient with blood and imaging tests as long as possible. Five years after the first medical consultation, the patient remained asymptomatic although the serum lipase and amylase values fluctuated widely. The amylase and lipase median values were 205.5 and 831.5 U/L, respectively (Figure 2). Interestingly, the lipase concentration never exceeded the value of 2,000 U/L. In the light of these observations, we hypothesized that hyperenzymemia in this situation may be considered as a true Gullo's syndrome case.

## 3. Discussion

In this report, we characterized the first case of Gullo's syndrome from Morocco. As biochemical screening of pancreatic hyperenzymemia in the patient's family members was negative, we considered that this reported case could be sporadic. Given the fact that clinical, radiological, and biological investigations were consistently normal for more than two years of follow-up, we suggested that the case we described fits perfectly with Gullo's syndrome. The possible pathological mechanism of this type of pancreas manifestation is still not clearly established. The variation in pancreatic enzyme concentrations detected in the peripheral blood could be attributed to a disruption of the cellular secretory mechanism. Such a disturbance would be due to an alteration of the basolateral surface of the acinar cells, leading to the secretion of enzymes in the peripheral blood rather than in the duodenum [8].

In addition, our patient has previously developed severe endometriosis disease. Sometimes, endometriosis disorders could be associated with aggressive manifestations during their development leading to other severe complications [4, 5]. Endometrial implants are often found in the rectum, sigmoid colon, lymph nodes, umbilicus, skin, bladder, kidney, and pancreas [8]. Lymphatic dissemination or blood vessels could be responsible for these unusual locations [9, 10]. As our patient presented endometriosis associated with severe symptoms, at first we thought that the pancreas may be a transportation target of endometrial fragments which could explain the observed pancreatic hyperenzymemia. Unfortunately, over five years of follow-up, no laparoscopy control exploring the course of this patient's endometriosis has been done. Given insufficient data on the location, extent, and size of endometrial implants in this patient, we therefore postulated that the occurrence of Gullo's syndrome and endometriosis could likely be just a coincidence. In the future, in-depth documentation of either elevation of pancreatic enzyme levels during the time of menses or the laparoscopic confirmation of extensive endometriosis in a large cohort of patients with endometriosis is needed to better understand this possible relationship.

According to the documented data, an apparently benign pancreatic hyperenzymemia can be the first clinical sign of a pancreatic tumor, especially in the elderly age group [11]. In our case, the regular monitoring over five years of follow-up showed that no malignancy sign of the pancreas was diagnosed. Nevertheless, highly accurate medical surveillance for a longer period is needed to confirm these findings.

## 4. Conclusion

Gullo's syndrome is a relatively new entity with a few reported cases worldwide. Our first reported case in Morocco is interesting and deserves careful attention to reassure the concerned patient that her condition is quite normal. It can also be helpful for physicians to avoid repeated tests and unnecessary treatments.

## Figures and Tables

**Table 1:** Complementary exams.

Indicators	Reference interval	2011^¥^	2016^#^
Fasting glucose (mg/dL)	72–110	0.77	0.78
Urea (mmol/L)	3.30–7.50	5.90	6.03
Creatinine (*µ*mol/L)	53–115	62	75
Total cholesterol (g/L)	1.16–2.40	2.30	2.29
Triglycerides (g/L)	0.35–1.59	1.20	1.01
Alkaline phosphatase (U/L)	53–115	72	58
Amylase (U/L)	30–110	**198**	**140**
Lipase (U/L)	27–280	**1461**	**527**
Gamma-GT (U/L)	8–78	14	17
Alanine transaminase (U/L)	5–35	25	22
Aspartate transaminase (U/L)	7–56	30	13
C-reactive protein (CRP) (mg/L)	<5	<5	<5
TSH (*µ*UI/mL)	0.35–4.96	2.72	2.11
Alpha-fetoprotein (AFP) (ng/ml)	<10	6.88	5.92
Carcinoembryonic antigen (CEA) (ng/ml)	<5	1.6	1.37
Carbohydrate antigen 19-9 (CA19-9) (U/ml)	<37	19.65	7.49
Carbohydrate antigen 125 (CA125) (IU/ml)	<35	5.49	4.90

^¥^Initial laboratory testing; ^#^last laboratory testing.
